# Improving child nutrition in disasters by developing a modeled disaster preparedness nutrition education curriculum

**DOI:** 10.3389/fpubh.2023.1293875

**Published:** 2023-12-07

**Authors:** Akindele Abimibayo Adeoya, Shinichi Egawa, Adebayo Sunday Adeoya, Ryoichi Nagatomi

**Affiliations:** ^1^Medicine and Science in Sports and Exercise Laboratory, Tohoku University Graduate School of Medicine, Sendai, Miyagi, Japan; ^2^Division for Interdisciplinary Advanced Research and Education, Tohoku University Advanced Graduate School, Sendai, Miyagi, Japan; ^3^International Cooperation for Disaster Medicine Laboratory, International Research Institute of Disaster Science (IRIDeS), Tohoku University, Sendai, Miyagi, Japan; ^4^Research and Development Division, Kerry Ingredients Nigeria Limited, Ikeja, Lagos State, Nigeria; ^5^Department of Biomedical Engineering for Health Maintenance and Promotion, Graduate School of Biomedical Engineering, Tohoku University, Sendai, Miyagi, Japan

**Keywords:** disasters, disaster preparedness nutrition education, malnutrition, student-centered curriculum, elementary school, pupils

## Abstract

In contemporary global society, largely because of climate change and other natural and human-induced hazards, disasters are an almost daily occurrence. The nutritional impact of disasters on children results in long-term physical and mental health problems. As children are one of the most vulnerable demographic groups, they must be empowered with disaster preparedness nutrition knowledge, and the skills and motivation to survive, prevent/reduce malnutrition, and maintain good health during disasters. A disaster preparedness nutrition education program (DPNEP) was developed in this study to improve children’s nutrition in daily life and during disasters through student-centered education. A consultative approach was used to synthesize the knowledge of a diverse group of four experts in disaster medicine and management, public health, education, and food and nutrition sciences to reach a consensus through discussion. A model DPNEP was developed by targeting grade 4 and 5 students and using interactive teaching methods. This can lead to the implementation of continuous nutrition education to empower children to make healthy food choices in daily life and reduce the risk of disaster-nutrition-related morbidity and mortality. Furthermore, once children acquire the necessary information, they are likely to share this knowledge with their families and communities, thereby enhancing society’s resilience.

## 1 Introduction

In contemporary global society, disasters are an almost daily occurrence, largely because of climate change and other natural and human-induced hazards. These disasters cause much misery, especially in non-resilient societies. The average frequency of disasters due to natural hazards worldwide was 13% higher in 2021 compared to that in 1991 (1), causing substantial economic losses. The Emergency Event Database recorded 387 global disasters, accounting for 30,704 deaths and affecting 185 million people, with economic losses amounting to approximately USD 223.8 billion (2). According to the World Health Organization, as of May 2023, over 766 million people had contracted COVID-19, with over 6.9 million fatalities (3). In addition to the costs associated with rising poverty, human costs have impacted global economic growth, disrupted lives and careers, and increased social unrest (4). The global disaster trends and impacts, 2001–2020 vs. 2021 and 2022, are summarized in Table 1.

**Table 1:** Global disaster trends and impacts, 2001–2020 vs. 2021 and 2022.

Disaster trends and impacts		Year	Total	Asia	Americas	Africa	Europe	Oceania
Frequency of disasters		2001–2020 (annual average)	347	-	-	-	-	-
		2021	432	174	129	57	56	16
		2022	387	137	118	79	43	10
Human Impacts	No. of deaths	2001–2020 (annual average)	61,212	62.7%	21.2%	3.7%	12.2%	0.2%
		2021	10,492	48.7%	43.2%	5.1%	2.9%	0.1%
		2022	30,704	24.7%	5.4%	16.4%	53.4%	0.1%
	Population affected in millions	2001–2020 (annual average)	193.4	84.5%	7.0%	8.0%	0.3%	0.2%
		2021	101.8	65.5%	4.6%	29.4%	0.4%	0.1%
		2022	185	34.6%	5.5%	59.6%	0.1%	0.2%
Economic losses (billion USD)		2001–2020 (annual average)	153.8	42.5%	45.5%	0.9%	8.4%	2.8%
		2021	252.1	18.9%	58.9%	0%	20.7%	1.4%
		2022	223.8	21.8%	69.6%	3.8%	0.1%	3.8%

The global disaster trends and impact data are sourced from the Emergency Events Database (EM-DAT) of the Centre for Research on the Epidemiology of Disasters (CRED), United States Agency for International Development (USAID), Université Catholique de Louvain. 2021, 2022 Disasters in Numbers (2, 5). This table represents disasters related to natural hazards, excluding biological and extra-terrestrial origins.

The challenges of ending hunger, food insecurity, and child malnutrition in all its forms continue to grow. Additionally, governments worldwide support food production and agriculture with an estimated USD 630 billion annually, but they have failed to deliver healthy diets to children and other vulnerable populations (6). In addition to other numerous accompanied hardships, disasters compound these difficulties for children, highlighting the global inequalities they face; moreover, they affect children’s nutritional status, dietary intake, and long-term development and cause general anthropometric failure (7). A longitudinal study in rural Nepal (8) demonstrated that despite a decrease in other infectious diseases during the COVID-19 pandemic, the pandemic exacerbated food insecurity and child malnutrition because of reduced employment opportunities, household income, and accessibility to and affordability of nutritious food, which further increased child vulnerability. The study emphasized the need for disaster preparedness with a focus on adequate nutrition, access to water, sanitation, hygiene, and healthcare supplies. Studies have indicated that undernutrition, particularly stunting, wasting, and underweight conditions, is rampant among children in flood-affected areas in low- and middle-income countries (9, 10). Effective preparedness for improving children’s nutrition during disasters must consider, respect, and leverage customs, cultural practices, religion, individual tolerance, psychological norms, education, and values. Cultural humility, awareness, and sensitivity are important in addressing the trauma that disasters inflict on children and young people (11, 12).

## 2 Rationale for disaster preparedness nutrition education

Damage from disasters can be worse when people lack preparedness and the ability to cope with hazards. Increased exposure to natural and human-induced hazards threatens people’s livelihoods and sustainable development efforts. Subsequently, the United Nations International Children’s Emergency Fund (UNICEF) and United Nations Educational, Scientific and Cultural Organization (UNESCO) have argued that education has an important role to play in preparing communities, saving lives, and building disaster-resilient societies (13). The Sendai Framework for Disaster Risk Reduction 2015–2030 emphasizes embedding disaster preparedness in daily life. It defines the goal of disaster education as the development of individual capacity based on the concept of the “three helps”: self-help, mutual help, and public help (14, 15). Torani et al. (16) also demonstrated that disaster education is a functional, operational, and cost-effective tool for risk management.

Nutrition security is a human right. Food and nutrition are important in fostering humanity in children and helping them acquire life skills (17). To stay healthy, people require basic knowledge of what constitutes a nutritious diet and how to best meet their nutritional needs from the available resources, including unhealthy food- and nutrition-related practices (18). Maintaining a healthy diet is essential in childhood because the eating habits that children learn early in life are often carried forward into adulthood. Providing an effective nutrition education program will help children build strong, healthy eating habits (19) and consider the concept of food security in daily life and during emergencies. Preparedness is the key to successful evacuation and is the most effective way to minimize damage during a disaster (20). Strong nutrition and disaster preparedness require the active participation of children and youth; food security should be a part of disaster preparedness to build children’s resilience and nutritional health and prevent/reduce negative long-term impacts of disasters (12, 21, 22).

Schools are crucial systems, play a pivotal role in helping students establish healthy eating behaviors (23), and are often designated to function as evacuation centers as well. Formal education can enhance disaster preparedness and reduce disaster vulnerability (24). Studies have indicated that school-based nutrition education programs increase children’s nutrition knowledge and promote healthy eating habits (25–30). Despite the effectiveness of nutrition education in promoting a healthy diet and the role of disaster education in increasing survival among children during disasters, malnutrition and malnutrition-related morbidity and mortality remain prevalent among children during disasters. This could be attributed to the absence of school-based education programs/interventions that integrate and simulate disaster-nutrition-related issues for children. According to Pérez-Rodrigo and Aranceta (31), nutrition education should be designed according to the needs and interests of students, teachers, and the school. Nutrition education that focuses on the younger generation, incorporates various lifestyles, extends healthy life expectancy, and considers the food cycle and environment to pass down healthy food culture to younger generations constitutes the essential basic policy for food and nutrition (32) as well as disaster preparedness. Children who are nutritionally disadvantaged and facing food insecurity during normal times will have further opportunities to understand nutritional scenarios through disaster preparedness nutrition education; therefore, this knowledge will also help prevent their worsening malnutrition.

Disaster preparedness nutrition education can be incorporated flexibly at different times in a regular school day, allowing schools to use strategies tailored to specific settings, daily schedules, and resources (23). Nutrition education is effective only when it is based on an adequate analysis of nutritional problems and provides a clear and concise definition of the objectives and methods of communication (18). In a review, Torani et al. (16) noted that planning and designing a comprehensive education program is necessary to cope with disasters, especially for children, pregnant women, older adults, and people with disabilities. An intervention study involving a participatory activity for primary school pupils, aimed at building community resilience, showed that children had a more positive view of their health and community food afterward (33). Similarly, following the August 2018 earthquake in Lombok Island, Indonesia, an integrated nutrition rehabilitation intervention, including health, nutrition, education, and care, positively impacted the growth and development of children under 5 years in post-disaster conditions (34). The soaring cases of infectious diseases, mental health issues, and malnutrition-related morbidity and mortality among children in disaster contexts, occasioned by food insecurity, pre-disaster malnutrition, sociocultural factors, and organizational challenges, emphasize the need for disaster preparedness nutrition education for children’s empowerment (12). This education creates awareness of the importance of prevention and preparedness, thereby bridging the gap between knowledge and action (35). It is also important to establish an education program and activities that enable children to adapt to and simulate expected nutritional challenges during disasters and devise a coping strategy for optimal health.

## 3 Study objective

This study aimed to develop a modeled educational program focused on improving children’s nutrition in daily life and during disasters, to enhance and provide schoolchildren with essential knowledge and practical skills vital for addressing and overcoming the nutritional challenges during disasters.

## 4 Principles underlying disaster preparedness nutrition education program (DPNEP)

Developing a pedagogical framework for children’s disaster preparedness nutrition education necessitates a combination of experiential learning and holistic strategies. Central to this framework lies experiential learning theory (36), which emphasizes active engagement through direct experiences and reflective practices. In the context of disaster preparedness and nutrition, this translates into hands-on activities, real-life simulations, and scenario-based role-playing. For instance, children can actively practice rationing and selecting nutritious food sources, followed by reflective discussions on their choices and potential outcomes. This approach deepens their comprehension of nutrition during disasters. Additionally, ecological systems theory (37) highlights the interconnectedness of various factors, urging educators to incorporate diverse environmental contexts into their teaching methods. Therefore, DPNEP should integrate considerations from the family, community, and broader society. This inclusive perspective ensures that children can apply their nutrition knowledge to different disaster scenarios and adapt effectively.

The whole child approach enriches this multifaceted perspective by emphasizing the importance of addressing a child’s physical, cognitive, and emotional domains (38, 39). This holistic approach encompasses nutritional choices, mental health, hygiene, and basic first aid to help learners internalize the multifaceted nature of disaster scenarios, ensuring that they not only survive but also thrive in post-disaster environments. Moreover, technology integration into the educational framework enhances the learning experience and promotes the understanding of complex concepts, especially for children. Interactive apps and simulations breathe life into abstract nutrition principles, allowing children to visualize and apply their knowledge to real-world disaster situations. A multifaceted approach rooted in experiential learning and extended by ecological understanding and technological integration provides a solid foundation for children’s disaster nutrition education in a student-centered manner. In addition, Sphere Project’s handbook, titled *Humanitarian Charter and Minimum Standards in Humanitarian Response* (40), can guide the program’s nutritional standards, ensuring practicality and adherence to global best practices.

## 5 Learning environment for DPNEP

Establishing an effective learning environment for a DPNEP entails integrating practical knowledge, interactive engagement, and emotional support. This unique program requires a setting that combines practical knowledge with engaging activities to ensure retention and application. The ideal environment prioritizes safety, active engagement, and relevance. Safe and comfortable spaces enhance learning (41). Active learning through hands-on activities, simulations, and role-playing can replicate real disaster situations and improve comprehension, aligning with the experiential learning advocated by Kolb (36). The use of visual aids, such as charts, videos, and diagrams, can simplify complex nutritional concepts and aid retention. This approach is consistent with multimedia learning theory (42), which highlights the benefits of synergies between visual and verbal information processing. Furthermore, ensuring that content is culturally appropriate is crucial for creating a familiar learning environment. The UNESCO Guide on Education for Sustainable Development Goals advocates context-relevant education, especially for disaster preparedness, empowering learners to address challenges by taking appropriate action (43).

Through the DPNEP, children will understand the significance of nutrition in maintaining optimal health, energy, and well-being, especially during emergencies. They will also recognize the essential nutrient-rich foods within and outside the community, which can be safely stored for long durations; understand the role of water and hygiene; and become familiar with the basic principles of food safety during and after a disaster. In addition, through hands-on activities and practical scenarios, they will be empowered to make healthy food choices in daily life and undertake safety decisions that will benefit them and their families, ensuring resilience and well-being during disasters. Lastly, beyond providing mere knowledge and teaching practical skills, the program will instill a sense of responsibility and empowerment in the children. They will be encouraged to advocate for proper nutrition at home and in their communities, understanding that their choices have a profound impact on not only their own health, but also the well-being of those around them during a disaster. Through this holistic approach, children will acquire important knowledge and be inspired to become proactive agents of change in their communities.

### 5.1 Monitoring and evaluation framework for DPNEP

The implementation of a “Monitoring and Evaluation Framework” is crucial for determining the effectiveness of DPNEP. At its core, the program’s goal is to endow students with actionable knowledge and practical skills to navigate nutritional challenges during disasters. The objectives of this program are multifold, aiming to enhance students’ understanding of safe food and nutrition and good hygiene practices; improve their ability to make informed food choices in daily life and during disasters; and instill essential food preparation and rationing skills while embedding a culture of preparedness. The program also seeks to influence the broader community culture of schools. Key performance indicators have been established to track and measure program outcomes, which have been categorized into knowledge, skill, behavioral, and cultural indicators. Knowledge indicators, for instance, include the ability of students to identify locally sourced safe food items and evacuation sites, and recognize good hygiene practices, while skill indicators focus on students’ proficiency in handwashing, food handling, and meal planning with emergency and limited supplies. Behavioral and cultural indicators assess student-led initiatives and the impact of the program on a community’s disaster preparedness measures. To gather relevant data, a combination of pre- and post-program surveys, skill demonstrations, parent questionnaires, and teacher observations are utilized, ensuring a robust and comprehensive data collection process.

**Figure 1:**
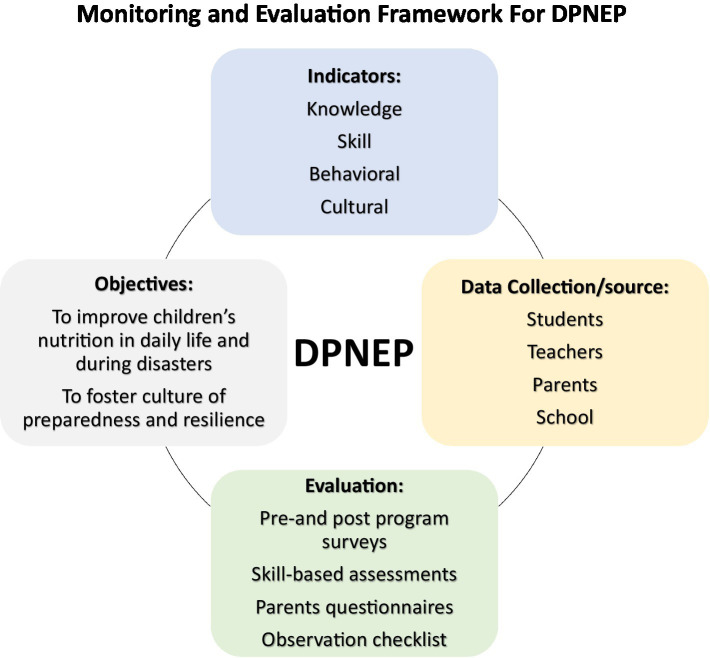
Monitoring and evaluation of framework for DPNEP.

The sources of data are diverse, ranging from student assessments and teacher feedback to parental insights on their child’s behavioral changes, and school records detailing participation and outcomes. This robust collection of data points enables a multi-dimensional evaluation of the effectiveness of DPNEP. Evaluations are strategically phased, with short-term evaluations assessing immediate program impact, intermediate evaluations monitoring knowledge retention and skill application after 3 months, and long-term evaluations analyzing sustained behavioral change and cultural integration on an annual basis. The framework emphasizes the importance of feedback for continuous improvement. Evaluation findings are disseminated to stakeholders, including school administrators, teachers, students, and parents, fostering a collaborative environment for program enhancement. Adjustments to the DPNEP are guided by this feedback, aiming to refine and optimize the program in accordance with the observed needs and success rates. Through such iterative refinement, the framework not only assesses the immediate impact of DPNEP but also ensures its long-term efficacy in preparing students with the critical knowledge and skills needed to handle nutritional challenges during disasters. The Monitoring and Evaluation Framework for DPNEP is illustrated in Figure 1.

## 6 Result to date/assessment process

Guided by the results from previous studies (12, 30), curricula for school-aged students were drafted for a DPNEP. A consultative approach was used to synthesize the knowledge of a group of diverse experts through discussion. The team consisted of four experts in disaster medicine and management (SE), public health (SE, RN), education (AAA), and food and nutrition sciences (ASA) with more than 5 years of professional practice/research experience. A draft of the “DPNEP for Daily Life and Emergencies,” containing the basic components listed in Table 2, was presented to each expert to comment on, modify, or make recommendations. After careful consideration and integration of the experts’ contributions, an updated version was developed. Subsequently, the team coordinator (AAA), an education expert, facilitated one or more live discussions via videoconferencing software and face-to-face sessions with individual participants for clarification, re-assessment, and consensus. The final version was developed and sent to each expert for confirmation and approval. The proposed “Disaster Preparedness Nutrition Education Program for Daily Life and Emergencies” is illustrated in Table 2.

**Table 2:** Proposed Program for the Disaster Preparedness Nutrition Education Program for Elementary Schools.

Target Students: Students in grades 4–5 in elementary school Style: Special subject with simulation play Duration: 30 min/class					
Topics/Contents	Objective	Methods	Key Message	Instructional Materials	Evaluation
Introduction: food, adequate diet, nutrients, safety, variety, enjoyable, social acceptance, household, community, and disaster	At the end of the class, participants should be able to give meaning to or get used to keywords.	Discussion	We need a variety of foods to grow and be healthy	Graph/audiovisual material illustrating food charts	What is food? What are nutrients?
Why we need food: To be healthy and well-nourished; for energy, growth, physical activity, and basic body functions	At the end of the class, participants should be able to establish what constitutes a good diet	Play/discussion	We need a variety of foods to grow and be healthy	Graph/audiovisual material illustrating the stages of child development	Why do we need food?
Nutrients and their functions: Carbohydrates, proteins, vitamins and minerals, fats, and oil	At the end of the class, participants should be able to explain the functions of the main nutrients	Discussion	Different groups have different dietary needs	Graph/audiovisual material illustrating the major classification of food	What are the functions of proteins? What are the functions of carbohydrates?
Food sources: Cereals (rice, wheat), root and tuber (carrots, sweet potato), pulses (beans, groundnuts), fruits (bananas, apples), meats, poultry, fish, milk, oil, fats (vegetable oil), and others (e.g., sugar)	At the end of the class, participants should be able to state the nutrient content of common foods	Storytelling	Different foods provide different nutrition	Graph/audiovisual material illustrating the major classification of food	Mention the food sources that you know
Dietary needs: The need for variety. Different groups have different dietary needs	At the end of the class, participants should be able to appreciate the need for variety in a diet and recognize individual dietary needs	Discussion and storytelling	A healthy diet can be simple and inexpensive	Graph illustrating the wide variety of locally available food	Mention the dietary needs of children
How to plan a mixed and balanced diet: Dietary guidelines (lifestyle, hygiene, and sanitation). Local and common diets (e.g., rice, wheat, maize, potatoes, and its accompaniments – fish, vegetables, soup or stews, and water)	At the end of the class, participants should know how to enrich a meal	Demonstration/project-based	A healthy diet can be simple and inexpensive	Graph illustrating the major classification of food and audiovisual materials	What is a balanced diet?
Malnutrition: Bad diet, malnutrition, undernutrition, overnutrition, hunger, and illness	At the end of the class, participants should be familiar with the keywords	Discussion and play	Malnutrition puts children at risk during disasters and affects learning	Graph illustrating a group of children suffering from nutrition-related diseases	What is malnutrition?
The causes of malnutrition: Poverty, food insecurity, disaster, poor health and sanitation, lack of knowledge and care	At the end of the class, participants should be able to recognize the causes of malnutrition	Storytelling/discussion	Malnutrition has numerous causes	Graph illustrating a group of children suffering from nutrition-related diseases	What are the causes of malnutrition?
Disasters and examples: A disaster is a sudden accident or natural tragedy that causes great damage or loss of life (e.g., earthquakes, typhoons, tsunamis, pandemics, wildfires, drought, thunderstorms)	At the end of the class, participants should be able to understand and list examples of disasters	Discussion/storytelling	A disaster causes great damage and loss of life	Graph illustrating examples of disasters. Disaster video clip	State examples of a disaster
Basic emergency survival kit: For example, water, food, flashlight, blanket, whistle, first aid kits, toiletries, waterproof case, and battery-powered radio	At the end of the class, participants should be able to identify emergency survival kits	Demonstration	Adequate preparedness saves lives	Graph and/or audiovisual materials illustrating an emergency survival kit	List the emergency kit items
Symbols of evacuation: Evacuation sites, evacuation centers, tsunami sites, evacuation routes, and basic school map (e.g., sports building, playground, cafeteria, toilet, school gate, signage, surrounding environment)	At the end of the class, participants should be able to recognize basic evacuation symbols (universal signage)	Excursion	Knowledge of evacuation symbols reduces danger and stress	Cards/audiovisual material illustrating evacuation symbols	Identify the evacuation symbols in your community
Strategies for fighting malnutrition during disasters: Action by communities and individuals and the role of nutrition education and school with emphasis on identification, innovation, and improvisation using locally available materials	At the end of the class, participants should be able to recognize strategies for fighting malnutrition during disasters, particularly the role of nutrition education	Discussion/project-based	Appropriate application of nutrition education can fight malnutrition in daily life and during disasters	Graph illustrating a group of children suffering from nutrition-related diseases	What is the role of the individual in fighting malnutrition?
Role of children during emergencies: For example, stay with, listen, and respond adequately to instructions from your guardian; inform your guardian of your needs, including food and allergies; maintain proper hygiene; advise/remind guardians of basic survival kits; raise the alarm if you suspect any danger; if alone, follow the evacuation route to the nearest evacuation center/sites; have consideration/empathy for others	At the end of the class, participants should be able to state their role during emergency	Discussion/roleplay	We must show empathy and respond adequately during emergencies	Audiovisual material illustrating emergencies	State the roles of children during a disaster

## 7 Discussion on the practical implication

The means to achieve sustainable solutions to child malnutrition in all its forms and circumstances is to educate and empower children and expand their life opportunities. When a child is nutritionally prepared through education to deal with multiple scenarios and complex emergencies, a ripple effect catalyzes far-reaching impacts on the community. The program content involves simple, understandable, and well-integrated disaster-nutrition messages and can be taught by academic staff in elementary schools with little or no training required. A student-centered curriculum considers learners’ needs, experiences, perspectives, abilities, interests, and backgrounds (44–46). Further, student-centered instruction enhances cooperative learning and enables students to make necessary decisions and develop independent problem-solving skills (47). The target population in this study consisted of students in grades 4 and 5 of elementary school. Students at this age are both young/cognitively flexible and old/cognitively sophisticated enough to prevent and reduce malnutrition in daily life and during disasters through acquired knowledge and skills actively and passively.

Moreover, they are not faced with the challenge of preparing for secondary school; therefore, targeting this group could encourage implementation in practice. Previous studies (30, 48) have demonstrated that the inclusion of 30 min of nutritional education per week in elementary school schedules is effective in improving healthy eating behavior; thus, it is important to provide nutrition education that integrates disaster preparedness into the formal education system to develop children’s abilities and preparedness for disasters. This will improve children’s understanding of nutrition in daily life and during disasters and provide opportunities for them to share what they have learned with their families, thereby promoting family and community understanding.

Our educational tool integrates disaster preparedness and nutrition education, increasing children’s motivation and skills and the opportunities for them to make informed decisions and engage in health-promoting actions, particularly regarding food choices and safety before, during, and after disasters. However, readers and practitioners should recognize that, while this program was developed based on experts’ recommendations and common best practices, it has not yet undergone a comprehensive validation process. As a result, the overall effectiveness, adaptability, and long-term impact of this program in real-world scenarios remains uncertain. This DPNEP may occasionally be validated in real disaster scenarios, but pre- and post-measurements of students’ knowledge, attitude, and practice can evaluate its value. The student-centered curriculum encourages active learning and increases content retention by providing practical, hands-on learning on healthy eating while promoting disaster preparedness. A special unit within a school health program can be employed alone or as part of subjects such as physical and health education, social studies, basic science, or agricultural science, depending on each school’s environment and resources. Schools can implement the model according to the needs of the school and the community. For instance, it could be an integral part of a camping activity to make it more attractive and fun for students or a picnic incorporating their families and the community. Schools should also identify and secure the available materials and resource persons such as the firefighters, waste management personnel, nutritionists, clinics, disaster management and public health experts, psychologists, gardens, chefs, market leaders, and experienced older adults within the community to facilitate learning, allowing students to become familiar with key facilities, equipment, locations, food, and persons in the community. During a disaster, this will allay or reduce fear and anxiety and help children maneuver their way to safety without compromising their nutritional health; this will make them better equipped to make informed decisions about food and water safety, potentially preventing illness or malnutrition during critical post-disaster periods.

Children’s knowledge can also serve as a safeguard against long-term health issues stemming from sustained food insecurity, ensuring they choose foods that provide essential nutrients. This knowledge becomes an invaluable asset in a landscape where malnourished children are increasingly susceptible to various diseases and developmental setbacks. Moreover, instilling such knowledge at a young age fosters a broader culture of preparedness within communities. This cascading effect reduces the immediate burden on relief agencies and strengthens community resilience, bolstering psychological well-being, fostering community cohesion through collective initiatives, promoting collective well-being, and promoting resource management. As these children mature, they carry this culture forward, ensuring that future generations are better prepared. While lack of empirical evidence, time factors, and school workload may affect the program’s implementation, it is intended to motivate students rather than merely serve as an evaluation tool. Through the precision education of the DPNEP for children, a more resilient, healthy, and sustainable future society is expected to emerge.

### 7.1 Limitations

Although built on a robust foundation of previous research on nutrition education for fourth- and fifth-grade students (30) and a scoping review of child nutrition during disasters (12), this study is not without its limitations. First, despite extensive background research, the current program did not have a specific pilot study tailored to its content and target population. This raises concerns about potential cognitive and developmental misalignments that may not adequately address the unique needs and levels of understanding of fourth- and fifth graders in the context of disaster preparedness. Second, although the program integrates instructional strategies that have proven effective in previous research, the specific level of interest and engagement of the target demographic in the context of disaster preparedness is untested. Their perceptions of this new content in terms of interest and internalization may differ from previous research findings on nutrition education in general.

Third, while the program is designed with flexible curriculum integration in mind and offers schools the adaptability to implement it based on their specific needs and that of the associated communities, the specific and logistical aspects of integration in a variety of school settings have not been empirically evaluated. This adaptive design acknowledges the importance of community- and school-specific requirements, but the nuances and unforeseen challenges of integration in diverse educational settings remain untested. In addition, this study may inadvertently introduce cultural and socioeconomic assumptions. Even with insights from prior research, the lack of specific pilot study implies that it remains an area of uncertainty whether this program will resonate with the diverse backgrounds and life experiences of all fourth- and fifth-grade students. Future iterations of this program and subsequent studies will benefit from direct empirical testing and feedback from the target population to further refine and optimize its effectiveness.

## 8 Conclusion

The DPNEP can lead to the implementation of continuous nutrition education to empower children to make healthy food choices in daily life and reduce the risk of disaster-nutrition-related morbidity and mortality. Furthermore, once children acquire the necessary information, they are likely to share this knowledge with their families and communities, thereby enhancing society’s resilience.

## Data availability statement

The original contributions presented in the study are included in the article/supplementary material, further inquiries can be directed to the corresponding author.

## Author contributions

AA: Conceptualization, Funding acquisition, Methodology, Resources, Validation, Visualization, Writing – original draft, Writing – review & editing. SE: Conceptualization, Methodology, Resources, Supervision, Validation, Visualization, Writing – review & editing. ASA: Conceptualization, Methodology, Resources, Validation, Visualization, Writing – review & editing. RN: Funding acquisition, Methodology, Resources, Supervision, Validation, Visualization, Writing – review & editing.
